# Inhaled Sargramostim (Recombinant Human Granulocyte-Macrophage Colony-Stimulating Factor) for COVID-19-Associated Acute Hypoxemia: Results of the Phase 2, Randomized, Open-Label Trial (iLeukPulm)

**DOI:** 10.1093/milmed/usac362

**Published:** 2022-12-02

**Authors:** Robert Paine, Robert Chasse, E Scott Halstead, Jay Nfonoyim, David J Park, Timothy Byun, Bela Patel, Guido Molina-Pallete, Estelle S Harris, Fiona Garner, Lorinda Simms, Sanjeev Ahuja, John L McManus, Debasish F Roychowdhury

**Affiliations:** Division of Respiratory, Critical Care and Occupational Pulmonary Medicine, University of Utah School of Medicine, Salt Lake City, UT 84132, USA; Department of Pulmonary and Critical Care, TidalHealth Peninsula Regional Medical Center, Salisbury, MD 21801, USA; Division of Pediatric Critical Care Medicine, Department of Pediatrics, Penn State University, Hershey, PA 17033, USA; Department of Medicine and Critical Care, Richmond University Medical Center, Staten Island, NY 10310, USA; Department of Hematology and Oncology, Providence St. Jude Medical Center, Fullerton, CA 92835, USA; Department of Hematology and Medical Oncology, Providence St. Joseph Hospital, Orange, CA 92868, USA; Department of Pulmonary, Critical Care and Sleep Medicine, University of Texas Health Science Center, Houston, TX 77030, USA; Department of Pulmonary and Critical Care, Great Plains Health, North Platte, NE 69101, USA; Division of Respiratory, Critical Care and Occupational Pulmonary Medicine, University of Utah School of Medicine, Salt Lake City, UT 84132, USA; Partner Therapeutics, Inc., Lexington, MA 02421, USA; Partner Therapeutics, Inc., Lexington, MA 02421, USA; Partner Therapeutics, Inc., Lexington, MA 02421, USA; Partner Therapeutics, Inc., Lexington, MA 02421, USA; Partner Therapeutics, Inc., Lexington, MA 02421, USA

## Abstract

**Introduction:**

Granulocyte-macrophage colony-stimulating factor (GM-CSF), a protein produced in the lung, is essential for pulmonary host defense and alveolar integrity. Prior studies suggest potential benefits in several pulmonary conditions, including acute respiratory distress syndrome and viral infections. This trial evaluated the effect of the addition of inhaled sargramostim (yeast-derived, glycosylated recombinant human GM-CSF) to standard of care (SOC) on oxygenation and clinical outcomes in patients with COVID-19-associated acute hypoxemia.

**Materials and Methods:**

A randomized, controlled, open-label trial of hospitalized adults with COVID-19-associated hypoxemia (oxygen saturation <93% on ≥2 L/min oxygen supplementation and/or PaO_2_/FiO_2_ <350) randomized 2:1 to inhaled sargramostim (125 mcg twice daily for 5 days) plus SOC versus SOC alone. Institutional SOC before and during the study was not limited. Primary outcomes were change in the alveolar–arterial oxygen gradient (P(A–a)O_2_) by day 6 and the percentage of patients intubated within 14 days. Safety evaluations included treatment-emergent adverse events. Efficacy analyses were based on the modified intent-to-treat population, the subset of the intent-to-treat population that received ≥1 dose of any study treatment (sargramostim and/or SOC). An analysis of covariance approach was used to analyze changes in oxygenation measures. The intubation rate was analyzed using the chi-squared test. All analyses are considered descriptive. The study was institutional review board approved.

**Results:**

In total, 122 patients were treated (sargramostim, *n* = 78; SOC, *n* = 44). The sargramostim arm experienced greater improvement in P(A–a)O_2_ by day 6 compared to SOC alone (least squares [LS] mean change from baseline [SE]: −102.3 [19.4] versus −30.5 [26.9] mmHg; LS mean difference: −71.7 [SE 33.2, 95% CI −137.7 to −5.8]; *P *= .033; *n* = 96). By day 14, 11.5% (9/78) of sargramostim and 15.9% (7/44) of SOC arms required intubation (*P *= .49). The 28-day mortality was 11.5% (9/78) and 13.6% (6/44) in the sargramostim and SOC arms, respectively (hazard ratio 0.85; *P *= .76). Treatment-emergent adverse events occurred in 67.9% (53/78) and 70.5% (31/44) on the sargramostim and SOC arms, respectively.

**Conclusions:**

The addition of inhaled sargramostim to SOC improved P(A–a)O_2_, a measure of oxygenation, by day 6 in hospitalized patients with COVID-19-associated acute hypoxemia and was well tolerated. Inhaled sargramostim is delivered directly to the lung, minimizing systemic effects, and is simple to administer making it a feasible treatment option in patients in settings where other therapy routes may be difficult. Although proportionally lower rates of intubation and mortality were observed in sargramostim-treated patients, this study was insufficiently powered to demonstrate significant changes in these outcomes. However, the significant improvement in gas exchange with sargramostim shows this inhalational treatment enhances pulmonary efficiency in this severe respiratory illness. These data provide strong support for further evaluation of sargramostim in high-risk patients with COVID-19.

## INTRODUCTION

The global COVID-19 pandemic has resulted in enormous morbidity and mortality, with over 6 million deaths.^1^ In severe cases, viral pneumonia progresses to respiratory failure and acute respiratory distress syndrome (ARDS).^2,3^ At its peak, mortality rates of up to 85% were reported in patients requiring intensive care. COVID-19 survivors often report long-term sequelae including persistent respiratory symptoms.^4^

Efforts to develop interventions for those with severe acute respiratory syndrome coronavirus 2 (SARS-CoV-2) infection have resulted in evidence-based therapies.^5–11^ However, these advances are limited and may not have shown unequivocal efficacy.^7^ Vaccination is protective against severe infection; however, incomplete acceptance of vaccination, incomplete protection for immunocompromised patients, and variant emergence highlight the ongoing need for the development of additional COVID-19 therapies.^8,11–13^ Ideally, new treatment options for COVID-19, and other respiratory viral illnesses, will be virus and variant independent, well tolerated, and easily administered.

Granulocyte-macrophage colony-stimulating factor (GM-CSF) plays a critical role in pulmonary host defense. GM-CSF drives functional maturation of alveolar macrophages,^14,15^ which are critical for lung health. Alveolar macrophages clear debris and pathogens and maintain surfactant homeostasis while limiting inflammation within the alveolar milieu.^16,17^ Additionally, through the maintenance of tissue-resident CD103^+^ dendritic cells, GM-CSF has a role in the initiation of pulmonary CD8^+^ T cell responses, as observed in mice.^18^ Furthermore, systemic opportunistic infections are a known phenomenon in patients with elevated GM-CSF autoantibodies in which the autoantibodies yield a deficiency in GM-CSF activity, highlighting the role of GM-CSF in host immune function.^19^ Of particular relevance here, mice that received anti-GM-CSF antibodies were more susceptible to SARS-CoV-2 infection and experienced decreased survival than phosphate-buffered saline-treated mice.^20^

Several studies demonstrated the ability of GM-CSF to rescue mice infected with lethal influenza A.^18,21,22^ Prior human studies reported recombinant human (rhu) GM-CSF as safe and well tolerated with potential benefit in patients with pulmonary conditions including ARDS and autoimmune pulmonary alveolar proteinosis (aPAP).^23–27^ In sepsis, GM-CSF is known to reverse immunoparalysis with beneficial outcomes.^28–30^ Additionally, sargramostim (yeast-derived, glycosylated rhu GM-CSF) can be administered directly to the lung by inhalation. In several clinical studies, including ARDS and aPAP, inhaled sargramostim use improved gas exchange and was well tolerated.^23–26^

Based on these mechanistic studies and prior patient experience, inhaled sargramostim was studied in patients hospitalized with COVID-19 who were hypoxemic, but not requiring ventilatory support, to investigate whether the addition of sargramostim to standard of care (SOC) would improve gas exchange and prevent disease progression.

## METHODS

### Study Design

This phase 2, randomized, open-label, interventional study investigated the efficacy and safety of sargramostim (yeast-derived, glycosylated rhu GM-CSF; Leukine^®^) in patients with COVID-19-associated acute hypoxemia (iLeukPulm). Although FDA approved for other indications with systemic administration, sargramostim is not authorized or approved for the treatment of COVID-19 or for administration via nebulization.^31^ The study was institutional review board approved (see additional details in Supplementary Methods). All patients or legal representatives provided written informed consent. A Data Safety Monitoring Board reviewed the data to ensure patient safety.

### Patients

Eligibility included hospitalized adult (≥18 years) patients with polymerase chain reaction–confirmed COVID-19-associated acute hypoxemia. Acute hypoxemia was defined as oxygen saturation of <93% on ≥2 L/min supplemental oxygen and/or the ratio of partial pressure of arterial oxygen to fraction of inspired oxygen (PaO_2_/FiO_2_) of <350. Patients requiring invasive mechanical ventilation, extracorporeal membrane oxygenation, or noninvasive support with continuous positive airway pressure (CPAP) or bi-level positive airway pressure (BiPAP) for hypoxemia at enrollment were excluded (full eligibility criteria in Supplementary Methods).

### Procedures

Patients were randomized 2:1 to receive inhaled sargramostim 125 mcg twice daily via mesh nebulizer for 5 days or until hospital discharge, if earlier, along with institutional SOC (sargramostim arm) or institutional SOC alone (SOC arm). The sargramostim dose was based on prior clinical study experience with improved gas exchange and minimal adverse events (AEs) in ARDS and aPAP.^23–26^ To prepare each dose, a 250-mcg sargramostim (Leukine^®^ [lyophilized powder] for injection) vial was reconstituted with 1 mL of preservative-free sterile water for injection to yield a clear, colorless solution. Then, 0.5 mL (125 mcg) of reconstituted sargramostim was withdrawn from the vial and further diluted with 1.5 mL of preservative-free 0.9% sodium chloride, which was then nebulized using a mesh nebulizer.

Eligible patients were required to be randomized within 48 hours of meeting study eligibility. Randomization was stratified by baseline sequential organ failure assessment score (<6 versus ≥6) and by investigational site using an interactive response technology system. Institutional SOC before and during the study was not limited and may have included supplemental oxygen, noninvasive and invasive ventilation (ventilatory support could only start after enrollment), antibiotics, antivirals, corticosteroids, and convalescent plasma. Patients were followed for 90 (±30) days for the assessment of clinical outcomes (additional details in Supplementary Methods).

As certain races and ethnicities have been identified as high-risk groups with regard to COVID-19 infection incidence and mortality,^32^ race and ethnicity data were collected for this study. Site staff selected the appropriate race and ethnicity, corresponding to the categories outlined in FDA Guidance.^33^ As needed, site staff may have asked patients to self-identify race and ethnicity.

### Outcomes

Because worsening hypoxemia is a key feature of COVID-19 clinical progression, we assessed the impact of sargramostim on change in the alveolar–arterial oxygen gradient (P(A–a)O_2_) by day 6 as a quantitative measure of gas exchange abnormality.^34,35^ Also, the need for intubation/mechanical ventilation by day 14 was a co-primary end point, reflecting COVID-19 progression. Secondary outcome measures included all-cause 28-day and 90-day mortality, treatment-emergent AEs, hospitalization duration, requirement for mechanical ventilation or extracorporeal membrane oxygenation (at any time during the study), supplemental oxygen duration, invasive ventilation duration, and ordinal scale change (ordinal scale and additional outcome details in Supplementary Methods).

### Statistical Analysis

This phase 2 study was designed at the onset of the global COVID-19 pandemic (March-April 2020) during which there were no authorized/approved COVID-19 treatments/vaccines. The sample size was based on practical and clinical considerations to ensure the evaluation of efficacy end points and safety profiles. As sargramostim demonstrated evidence of clinical activity and safety in aPAP, ARDS, and sepsis, it was hypothesized that inhaled sargramostim would also improve gas exchange and clinical outcomes in COVID-19. Because of no COVID-19-specific clinical data, the initial study design evaluated efficacy and safety in 40 patients treated with inhaled sargramostim and 20 patients with SOC alone. As the pandemic continued and safety data with inhaled sargramostim in COVID-19 was obtained and assessed by the Data Safety Monitoring Board, the study was amended to enroll 80 patients treated with inhaled sargramostim and 40 patients with SOC. The inclusion of additional patients enabled better precision of efficacy and safety end points.

Safety analyses were based on the treated population (received ≥1 sargramostim dose or SOC). Efficacy analyses were based on the modified intent-to-treat population, the subset of the intent-to-treat population that received ≥1 dose of any study treatment (sargramostim and/or SOC). There was no alpha control or multiplicity adjustment on any comparisons. All analyses are considered descriptive (analyzed using SAS Version 9.4).

The analytic study period began at the date/time of the sargramostim first dose. For patients who received SOC alone, randomization date/time was used as the analytic period start (additional details in Supplementary Methods). Up-to-day 6 evaluation was the main time point for the evaluation of oxygenation, based on arterial blood gas (ABG) measurements. The last ABG obtained before day 6 after enrollment was used to assess change in oxygenation.

For statistical analyses of changes in oxygenation measures, a mixed model repeated measures approach was planned. However, most patients had only baseline and up-to-day 6 evaluation; therefore, an analysis of covariance approach was used, in which change in P(A–a)O_2_ from baseline to up-to-day 6 was included as a dependent variable, treatment as a fixed effect, and baseline value as a continuous covariate. To further characterize improvement in gas exchange, the percentage of patients with ≥33% improvement or ≥50% improvement in P(A–a)O_2_ for each treatment arm was compared between arms using the chi-squared test. The intubation rate was analyzed using the chi-squared test. Time-to-event end points were estimated using the Kaplan–Meier methods, and the treatment effect was examined via log-rank test and hazard ratios (HRs) estimated from the Cox proportional hazards model. Categorical end points are calculated as the percentage of patients with the event, relative to the number of patients in a given analysis population. Continuous end points are summarized by *n*, means, medians, minimum, maximum, and 25th and 75th percentiles.

## RESULTS

**FIGURE 1.**
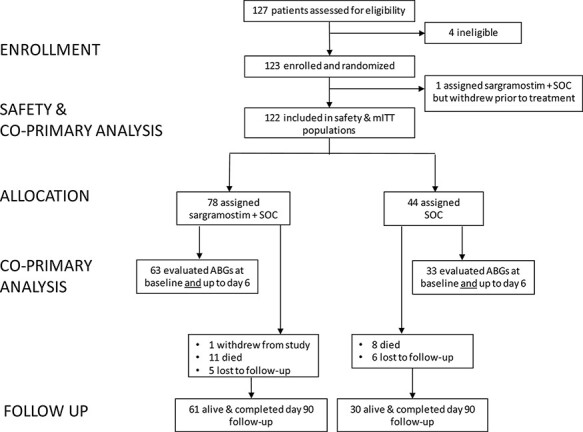
Eligibility, randomization, and follow-up of patients in the iLeukPulm study. Abbreviations: ABG* = *arterial blood gas; mITT* = *modified intention to treat; SOC* = *standard of care.

Between August 19, 2020, and February 17, 2021, 123 patients were randomized across 11 US centers (Fig. 1; list available in Supplementary Methods). Of 79 patients randomized to receive sargramostim, 78 (99%) received at least one dose. All patients received sargramostim within 1 calendar day of randomization. Of note, 29 patients (37%) did not receive a full 5 days of sargramostim because of the following: 3 patients (4%) discontinued treatment because of AE (described in more detail in the Safety subsection), 18 patients (23%) were discharged before day 5, and 8 patients (10%) missed ≥1 dose. Of the 44 patients who received SOC, 8 (18%) discontinued treatment before day 5. Of these, 7 (16%) were discharged, and 1 (2%) was transferred to another facility. The last patient follow-up occurred on May 19, 2021.

Patient baseline characteristics are detailed in Table I. The mean age was 60.4 years, with one-third of patients aged ≥65 years old. Half of patients were male. Two-thirds (66.4%) of subjects were white, and 5.7% were black/African-American; 28.7% were Hispanic/Latino. The mean body mass index was 35.1 kg/m^2^, and 69% of patients were obese. At baseline, the majority of patients reported hypertension (67%) and disorders related to glucose metabolism (62%), whereas 41% reported disorders related to lipid metabolism. A large percentage of patients reported respiratory, thoracic, and mediastinal disorders described in the Medical Dictionary for Regulatory Activities System Organ Class (58%) (e.g., respiratory failure [23%], asthma [17%], and sleep apnea [16%]).

**TABLE I.** Patient Baseline^a^ Characteristics and Other Treatments During Study

Characteristics	Sargramostim arm (*n = *78)	SOC arm (*n = *44)	Total (*N = *122)
Age at randomization, mean (SD), years	61.7 (13.0)	58.2 (10.6)	60.4 (12.3)
Age ≥65 years, *n* (%)	31 (39.7)	14 (31.8)	45 (36.9)
Sex, M, *n* (%)	35 (44.9)	26 (59.1)	61 (50.0)
Race, *n* (%)			
American Indian or Alaska Native	2 (2.6)	0	2 (1.6)
Asian	2 (2.6)	0	2 (1.6)
Black or African-American	6 (7.7)	1 (2.3)	7 (5.7)
Native Hawaiian or other Pacific Islander	0	1 (2.3)	1 (0.8)
White	48 (61.5)	33 (75.0)	81 (66.4)
Others	20 (25.6)	9 (20.5)	29 (23.8)
Ethnicity, *n* (%)			
Hispanic/Latino	24 (30.8)	11 (25.0)	35 (28.7)
Patients assessed for BMI at baseline, *n*	73	43	116
BMI, mean (SD), kg/m^2^	34.9 (9.0)	35.4 (9.6)	35.1 (9.2)
Obesity (BMI ≥30), *n* (%)	49 (67.1)	31 (72.1)	80 (69.0)
Baseline comorbidity (reported medical history)			
Hypertension, *n* (%)	53 (67.9)	29 (65.9)	82 (67.2)
Disorders related to glucose metabolism (diabetes mellitus, hyperglycemia, impaired glucose tolerance), *n* (%)	47 (60.3)	19 (43.2)	66 (62.3)
Diabetes mellitus^b^	36 (46.2)	15 (34.1)	51 (41.8)
Disorders related to lipid metabolism	36 (46.2)	14 (31.8)	50 (41.0)
Hyperlipidemia, *n* (%)	33 (42.3)	13 (29.5)	46 (37.7)
Dyslipidemia, *n* (%)	3 (3.8)	1 (2.3)	4 (3.3)
Respiratory, thoracic, and mediastinal disorders, *n* (%)	52 (66.7)	19 (43.2)	71 (58.2)
Asthma	16 (20.5)	5 (11.4)	21 (17.2)
Respiratory failure^c^	19 (24.4)	9 (20.5)	28 (23.0)
Sleep apnea syndrome	15 (19.2)	5 (11.4)	20 (16.4)
Baseline SOFA score, mean (SD)	2.3 (0.6)	2.4 (1.0)	2.4 (0.8)
Baseline ordinal score, *n* (%)^d^			
4	40 (51.3)	22 (50.0)	62 (50.8)
5	38 (48.7)	22 (50.0)	60 (49.2)
Baseline oxygenation, *n*	71	41	112
PaO_2_/FiO_2_ ratio, mean (SD)	146.8 (79.3)	132.8 (61.1)	141.7 (73.2)
P(A–a)O_2_, mean (SD), mmHg	293.8 (181.5)	322.1 (175.1)	304.2 (178.9)
Other COVID-directed treatments at any time during the study^e^			
Systemic corticosteroid, *n* (%)	72 (92.3)	43 (97.7)	115 (94.3)
Remdesivir, *n* (%)	59 (75.6)	36 (81.8)	95 (77.9)
Convalescent plasma, *n* (%)	13 (16.7)	10 (22.7)	23 (18.9)
Baricitinib	2 (2.6)	2 (4.5)	4 (3.3)
Tocilizumab	2 (2.6)	1 (2.3)	3 (2.5)
Hydroxychloroquine	0	1 (2.3)	1 (0.8)

Abbreviations: BMI = body mass index; FiO_2_* = *fraction of inspired oxygen; M = male; PaO_2_* = *partial pressure of oxygen; P(A–a)O_2_* = *alveolar–arterial oxygen gradient; SOC* = *standard of care; SOFA* = *sequential organ failure assessment.

a Baseline is defined as the last assessment before administration of study drug for sargramostim, or up to and including the randomization date for the SOC arm.

b “Diabetes mellitus” includes preferred terms “Diabetes mellitus,” “Type 1 diabetes mellitus,” “Type 2 diabetes mellitus,” “Insulin-requiring type 2 diabetes mellitus,” and “Diabetic complication/type 2 diabetes mellitus with complications.”

c “Respiratory failure” includes preferred terms “Respiratory failure,” “Acute respiratory failure,” and “Chronic respiratory failure.”

d Ordinal scores are based on a 9-point scoring system, ranging from 0 (no hospitalization) to 8 (death), where 4 is defined as “Hospitalized, requiring supplemental oxygen by mask or nasal prongs” and 5 is defined as “Hospitalized, requiring high-flow oxygen therapy, noninvasive mechanical ventilation, or both” (additional details on the ordinal scale in Supplementary Methods). Of note, noninvasive ventilation (CPAP, BiPAP, for hypoxemia) was an exclusion criterion for this study.

e Institutional SOC before and during the study was not limited and may have included antibiotics, antivirals, corticosteroids, and convalescent plasma. Other drugs/classes received by >5% of patients included azithromycin (11.5%) and doxycycline (13.1%). One patient on the sargramostim arm received a COVID-19 vaccine. One patient on the SOC arm received hydroxychloroquine.

Our patient population suffered severe SARS-CoV-2 pulmonary involvement as reflected by 49.2% of patients requiring high-flow oxygen at enrollment. Baseline mean ± SD P(A–a)O_2_ was 293.8 ± 181.5 mmHg (39.1 ± 24.1 kPa) on the sargramostim arm and 322.1 ± 175.1 mmHg (42.8 ± 23.3 kPa) on the SOC arm. Baseline mean ± SD PaO_2_/FiO_2_ was 146.8 ± 79.3 mmHg (19.5 ± 10.5 kPa) on the sargramostim arm and 132.8 ± 61.1 mmHg (17.7 ± 8.1 kPa) on the SOC arm. During the study, patients received other therapies to treat COVID-19 as part of SOC including 94.3% of patients received systemic corticosteroid therapy, 77.9% received remdesivir, and 18.9% received convalescent plasma (see additional details in Table I).

### Primary Outcomes

The analysis of change from baseline P(A–a)O_2_ was based on 96 of the total 122 patients with both baseline and up-to-day 6 blood gas measurements (see Table II; Supplementary Table S1 details the assessment distribution by arm). On the sargramostim arm (*n = *63), the mean change ± SE in P(A–a)O_2_ from baseline to up-to-day 6 was −102.3 ± 19.4 mmHg (−13.6 ± 2.6 kPa) versus −30.5 ± 26.9 mmHg (−4.1 ± 3.6 kPa) on the SOC arm (*n = *33) as assessed by least squares (LS) mean (LS mean difference ± SE: −71.7 ± 33.2 mmHg [−9.5 ± 4.4 kPa], 95% CI −137.7 to −5.8 mmHg [−18.3 to −0.8 kPa]; *P* = .033). The mean percent change from baseline was −30.9% and −5.2% for the sargramostim and SOC arms, respectively. More patients on the sargramostim arm had ≥50% improvement in P(A–a)O_2_ compared to the SOC arm (*P* = .039); although not statistically significant, proportionally more sargramostim-treated patients showed ≥33% improvement (*P* = .22) (Table II). Supplementary Figure S1 shows the change in P(A–a)O_2_ from baseline to up-to-day 6 measurement for each patient. On the sargramostim arm, 11.5% (*n = *9/78 patients) required invasive ventilation by day 14 versus 15.9% (*n = *7/44) on the SOC arm (*P = *.49).

**TABLE II.** Primary Outcomes

Parameter	Sargramostim arm	SOC arm	*P*-value
P(A–a)O_2_, patients with both baseline and post-baseline, up-to-day 6, *n*	**63**	**33**	
Baseline P(A–a)O_2_, mean (SD), mmHg	299.4 (183.5)	326.0 (172.2)	
Post-baseline P(A–a)O_2_, up-to-day 6, mean (SD), mmHg	199.5 (196.3)	290.8 (212.5)	
Change from baseline P(A–a)O_2_, up-to-day 6, LS mean (SE), mmHg	**−102.3 (19.4)**	**−30.5 (26.9)**	**.033**
LS mean (SE) difference, mmHg	−71.7 (33.2)		
95% CI of LS mean difference, mmHg	−137.7 to −5.8		
Percentage change from baseline P(A–a)O_2_, up-to-day 6, mean (SD)^a^	−30.9 (72.0)	−5.2 (91.0)	
Patients with improvement of P(A–a)O_2_			
Any improvement, *n* (%)	53 (84.1)	21 (63.6)	.023
≥50% improvement, *n* (%)	33 (52.4)	10 (30.3)	.039
≥33% improvement, *n* (%)	37 (58.7)	15 (45.5)	.22
Patients with intubation (mechanical ventilation) by day 14, *n/N* (%)	**9/78 (11.5)**	**7^b^/44 (15.9)**	**.49**

Abbreviations: ECMO* = *extracorporeal membrane oxygenation; LS* = *least squares; P(A–a)O_2_* = *alveolar–arterial oxygen gradient; SE = standard error; SOC* = *standard of care.

Primary outcomes are in bold.

a The percent change in P(A–a)O_2_ was calculated for each patient as the change from baseline divided by the baseline value. Then, summary statistics were generated.

b Two patients also received ECMO.

### Secondary Outcomes

All-cause 28-day mortality was 11.5% on the sargramostim arm versus 13.6% on the SOC arm (HR 0.85; 95% CI 0.30–2.39; *P = *.76; Table III). Through day 90, all-cause mortality was 14% and 18% of patients, respectively (HR 0.77; 95% CI 0.31–1.92; *P *= .58). There was no difference in the duration of hospitalization between arms. The duration of intensive care unit admission and the duration of invasive ventilation for those who required intubation were shorter on the sargramostim arm compared to the SOC arm; however, these differences did not achieve statistical significance. There was no difference in ordinal scores between arms from baseline to days 6, 28, or 90. See additional results in the Supplementary Results section.

**TABLE III.** Secondary Clinical Outcomes and Adverse Events

Parameter	Sargramostim arm (*n* = 78)	SOC arm (*n* = 44)	*P*-value
28-day mortality, *n* (%)	9 (11.5)	6 (13.6)	
Hazard ratio (95% CI)	0.85 (0.30 to 2.39)		.76
90-day mortality, *n* (%)	11 (14.1)	8 (18.2)	
Hazard ratio (95% CI)	0.77 (0.31 to 1.92)		.58
Duration of hospitalization, *n*^a^^,^^b^	78	44	
Mean (SD), days	12.1 (9.4)	11.8 (8.6)	–
Median (IQR), days	9.0 (6.0, 15.0)	9.0 (5.5, 14.5)	–
Discharged home	58 (74.4)	31 (70.5)	.64
Duration of ICU stay, *n*^b^	13	9	
Mean (SD), days	10.9 (11.9)	14.4 (12.7)	–
Median (IQR), days	6.0 (3.0, 12.0)	9.0 (4.0, 19.0)	–
Duration of invasive ventilation (ventilator or EMCO), *n*^b^	12	9	
Mean (SD), days	16.4 (14.4)	22.7 (23.8)	.46
Median (IQR), days	12.5 (9.5, 17.5)	19.0 (6.0, 26.0)	–
Ordinal score			
Baseline, *n*	78	44	
Mean (SD)	4.5 (0.50)	4.5 (0.51)	–
Day 6 change from baseline, *n*	58	33	
LS, mean (SE)	−0.1 (0.10)	−0.2 (0.14)	.46
Day 28 change from baseline, *n*	61	25	
LS, mean (SE)	−2.7 (0.15)	−2.7 (0.22)	.93
Day 90 change from baseline, *n*	61	30	
LS, mean (SE)	−3.3 (0.16)	−3.4 (0.23)	.81
Treatment-emergent adverse events, *n* (%)	53 (67.9)	31 (70.5)	–
Treatment-emergent serious adverse events, *n* (%)	15 (19.2)	14 (31.8)	–

Abbreviations: ECMO* = *extracorporeal membrane oxygenation; ICU = intensive care unit; IQR = interquartile range; LS* = *least squares; P(A–a)O_2_* = *alveolar–arterial oxygen gradient; SE = standard error; SOC* = *standard of care. Patients evaluated at both baseline and post-baseline time points are included in the table. A number of patients evaluated are noted for each outcome.

a Duration of hospitalization includes ICU days.

b All durations were assessed up to 90 (±30) days of follow-up.

### Safety

#### Inflammatory markers

Changes from baseline for markers of inflammation (ferritin, D-dimer, and C-reactive protein) by day 6 were similar between arms (Supplementary Table S2). No clinically meaningful differences were noted between study arms in any other reported hematologic or serum chemistry laboratory parameters (Supplementary Results).

#### Adverse events

Sargramostim was well tolerated with no differences in the proportions of patients with treatment-emergent AEs (67.9% [*n = *53] sargramostim arm versus 70.5% [*n = *31] SOC arm; Table III). Sargramostim-treated patients were less likely to experience treatment-emergent serious AEs (19.2% [*n = *15] sargramostim arm versus 31.8% [*n = *14] SOC arm), grade 3 and/or 4 treatment-emergent AEs (23.1% [*n = *18] sargramostim arm versus 29.5% [*n = *13] SOC arm), and fatal treatment-emergent AEs (14.1% [*n* = 11] sargramostim arm versus 18.2% [*n* = 8] SOC arm). Five patients (6.3%) had sargramostim-related treatment-emergent AEs (grade 1 [dry mouth, *n* = 1; hyponatremia, *n* = 1; cough, *n* = 1; chest discomfort and throat irritation, *n* = 1], grade 3 [hypoxia, *n* = 1]). Notably, no serious AEs were related to sargramostim.

Three sargramostim-treated patients (3.8%) had AEs resulting in discontinued treatment (hypoxia, *n* = 1; diarrhea, *n* = 1; nightmare and insomnia, *n* = 1). Of these, only a hypoxia event was considered possibly related to sargramostim by investigators. The patient’s oxygenation levels continued to fluctuate for several days after sargramostim discontinuation, with resolution of hypoxia after 18 days.

## DISCUSSION

The results of this randomized, controlled trial show inhalation treatment with sargramostim resulted in a greater improvement in oxygenation as measured by P(A–a)O_2_ in hypoxemic, hospitalized patients with COVID-19 compared to SOC alone. Those who received inhaled sargramostim experienced approximately three times greater improvement in oxygenation compared to patients receiving SOC alone, as measured by change in P(A–a)O_2_ (*P = *.033). For the second primary end point of percentage of patients requiring invasive mechanical ventilation by day 14, this study showed a modest but not statistically significant numerical improvement in the percentage of patients requiring invasive mechanical ventilation by day 14 (sargramostim arm versus SOC arm: 11.5% versus 15.9%; *P = *.49). Treatment with sargramostim was well tolerated.

The study population had high-risk features for severe COVID-19 disease, including older age, Hispanic or Latino ethnicity, obesity, hypertension, diabetes mellitus, and underlying pulmonary diseases.^8,32,36,37^ While male sex and obesity were proportionately more common on the SOC arm, all other known risk factors for COVID-19 mortality were higher on the sargramostim arm.^38^ Patients entering the study had severe respiratory compromise, indicated by high baseline P(A–a)O_2_ values and high levels of baseline oxygen support. Importantly, the patient population received SOC treatments (at that time point in the pandemic) for COVID-19 including corticosteroids, remdesivir, and convalescent plasma, which were balanced between the two arms.

While designed at the onset of the global COVID-19 pandemic (March-April 2020) during which there were no authorized/approved COVID-19 treatments/vaccines, this study remains relevant even as new treatments/vaccines have been developed. COVID-19 remains a significant cause of morbidity because of hypoxemia, hospitalization, long-term sequalae, as well as death in high-risk populations.^1,11^ Most disease-specific treatments developed to date (antivirals and monoclonal antibodies) are virus- and/or variant-specific.^7,8,11^ Non-virus-specific therapies (e.g., systemic corticosteroids) are principally utilized in hospitalized patients and often require intravenous administration.^5–7^ Recently, non-virus-specific antivirals have been developed.^10^ There is still a need for therapies, such as sargramostim, that are variant agnostic, easily administered, and widely available in a variety of settings.

SARS-CoV-2 infection could lead to significant pulmonary cellular destruction, which may be exacerbated by exuberant, dysregulated inflammation.^39,40^ Together these processes result in extensive alveolar exudates and ventilation–perfusion abnormality. In severe cases, shunt physiology leads to refractory hypoxemia and ARDS.^2^ Sargramostim may alter this progression of SARS-CoV-2 infection and other viral infections (e.g., influenza) through improvement in oxygenation by several different mechanisms. It may induce functional maturation of recruited mononuclear cells into alveolar macrophages, which enhance clearance of alveolar debris without stimulating excess inflammation.^22,41^ Sargramostim may also enhance viral clearance and may stimulate the proliferation of type II alveolar epithelial cells to restore alveolar wall integrity.^18,22,41^ These effects would all be anticipated to be maintained even if the virus mutates to avoid adaptive immune defenses.

There are several important features of our experimental design. Sargramostim was administered directly into the lung via nebulization. This route directs treatment to the site where endogenous GM-CSF acts to defend alveolar integrity. It also limits systemic exposure to sargramostim, potentially decreasing any theoretical contribution to pathologic systemic inflammation. Nebulized treatment is relatively noninvasive and simple to administer; thus, it may be feasible even in environments in which intravenous treatment may be challenging. Change in P(A–a)O_2_ was selected as one of the co-primary end points. Impaired gas exchange is a cardinal feature of severe COVID-19. P(A–a)O_2_ is a quantitative measure of lung dysfunction. Improvement in P(A–a)O_2_ is an objective indication of improved pulmonary function. Although this parameter is an imperfect surrogate for clinical benefits such as mortality, the significant improvement in gas exchange with sargramostim demonstrates that this inhalational treatment enhances pulmonary efficiency in this severe respiratory illness. Larger studies are required to formally test for a reduction in intubation or mortality.

Sargramostim is widely available with much clinical experience since FDA approval in 1991.^31^ Sargramostim is FDA approved for administration via the subcutaneous and intravenous routes and is not authorized or approved for the treatment of COVID-19.^31^ In this trial of patients with COVID-19, inhaled sargramostim was well tolerated with no AE increase compared to SOC alone. One patient was discontinued from sargramostim because of a hypoxic event that was considered possibly related to sargramostim by the investigators. Serious AEs and grade 3 and/or 4 treatment-emergent AEs were numerically lower on the sargramostim arm. Concerns that GM-CSF itself may contribute to hyperinflammation or cytokine storm in COVID-19 motivated trials of neutralizing antibodies to GM-CSF.^42^ Anti-GM-CSF antibodies have not shown unequivocal benefit. Furthermore, despite these theoretical concerns, no evidence of increased inflammation or cytokine storm was apparent in this trial, as measured by the number of AEs or increases in ferritin, D-dimer, and C-reactive protein levels.

This study was designed to evaluate and assess the efficacy and safety of inhaled sargramostim in patients with COVID-19, a disease for which inhaled sargramostim was not previously evaluated. This study was not designed or powered to show a significant difference in clinical end points such as mortality and duration of hospitalization. The sample size was based on practical and clinical considerations to enable better precision of estimates of efficacy and safety measures and evaluation of less common AEs. Limitations of this study include lack of blinded placebo control and missing ABG measurements for the estimation of P(A–a)O_2_. When this study was designed, there were great concerns about risk of aerosol therapies for spread of infection to hospital personnel. Based on this consideration, like many other studies, this study had a control group of participants receiving SOC, without a nebulized placebo. P(A–a)O_2_ is an objective measure of oxygenation. However, paired ABGs were not obtained in all patients between baseline and the day 6 time point. Of 122 patients, 96 (79%) had both baseline and post-baseline assessments for P(A–a)O_2_ despite this protocol requirement. This situation may in part be a function of less standard ABG utilization in recent years and was likely exacerbated by clinical system and staff overload from the number of COVID-19 patients requiring hospitalization at the height of the pandemic. It may also be reflective of clinical improvement that did not necessitate follow-up assessment. Although proportionally lower rates of intubation and mortality were observed in sargramostim-treated patients, this study was insufficiently powered to demonstrate significant changes in these outcomes, especially in light of the modest rates of intubation and death observed.

## CONCLUSIONS

This study provides evidence that inhaled sargramostim treatment for up to 5 days appears to improve P(A–a)O_2_, a measure of oxygenation, by day 6 in hypoxemic, hospitalized patients with COVID-19. Sargramostim therapy was well tolerated with no AE increase compared to SOC alone. The strong mechanistic underpinnings, good safety profile, and central physiologic importance of the impact on gas exchange support that inhaled sargramostim should continue to be investigated in COVID-19-associated acute hypoxemia and in other viral infections causing acute respiratory failure.

## Supplementary Material

usac362_Supp

## Data Availability

The data underlying this article will be considered for sharing upon reasonable request to the corresponding author.

## CLINICAL TRIAL REGISTRATION NUMBER

The trial was registered with ClinicalTrials.gov (NCT04411680) on May 29, 2020, with E. Scott Halstead, MD, PhD as the principal investigator.

## INSTITUTIONAL CLEARANCE

Institutional clearance does not apply.
