# Supplementary material for: Inhaled Sargramostim (Recombinant Human Granulocyte-Macrophage Colony-Stimulating Factor) for COVID-19-Associated Acute Hypoxemia: Results of the Phase 2, Randomized, Open-Label Trial (iLeukPulm)
Source: Mil Med. 2022 Dec 2;188(7-8):e2629–38. doi: 10.1093/milmed/usac362 (PMC10363010; doi:10.1093/milmed/usac362)
Supplement: usac362_Supp [file usac362_supp.zip › iLP_MilMed_Supplement_revised20221020_clean.docx]

**Supplemental Materials**

**Inhaled Sargramostim (Recombinant Human Granulocyte-Macrophage Colony-Stimulating Factor) for COVID-19-Associated Acute Hypoxemia: Results of the Phase 2, Randomized, Open-Label Trial (iLeukPulm)**

Robert Paine III, MD; Robert Chasse, MD; E. Scott Halstead, MD, PhD; Jay Nfonoyim, MD; David J. Park, MD; Timothy Byun, MD; Bela Patel, MD; Guido Molina-Pallete, MD; Estelle S. Harris, MD; Fiona Garner, PhD; Lorinda Simms, MSc, PStat, RAC(US); Sanjeev Ahuja, MD; John L. McManus; Debasish F. Roychowdhury, MD

**Table of Contents**

[**Supplementary Methods** 3](#_Toc108090165)

[Study design 3](#_Toc108090166)

[Key Eligibility Criteria 3](#_Toc108090167)

[*Inclusion Criteria* 3](#_Toc108090168)

[*Exclusion Criteria* 4](#_Toc108090169)

[Procedures 4](#_Toc108090170)

[Outcomes 5](#_Toc108090171)

[*P(A-a)O_2_* 5](#_Toc108090172)

[*Ordinal Scale* 5](#_Toc108090173)

[*Additional Planned Outcomes Not Reported* 6](#_Toc108090174)

[Statistical Analysis 7](#_Toc108090175)

[**Supplementary Results** 7](#_Toc108090176)

[Duration of Supplemental Oxygen 7](#_Toc108090177)

[Hematologic Data 8](#_Toc108090178)

[Table S1: Availability of P(A-a)O_2_ Assessments by Study Day 8](#_Toc108090179)

[Table S2: Safety Parameters for Markers of Inflammation 9](#_Toc108090180)

[Figure S1: Percent Change in P(A-a)O_2_ from Baseline (Y-axis) to Up-to-day 6 Evaluation for Each Patient (X-axis) 10](#_Toc108090181)

# **Supplementary Methods**

## **Study design**

The Western Institutional Review Board (20201524) and the US Army Medical Research and Development Command (USAMRDC) Office of Research Protections (ORP) Human Research Protection Office (HRPO) approved the study (A-21108.1a).

The work was performed in the following institutions: St. Jude Medical Center, Fullerton, CA, USA; St. Joseph Hospital of Orange, Orange, CA, USA; California Pacific Medical Center - Van Ness Campus, San Francisco, CA, USA; TidalHealth Peninsula Regional Medical Center, Salisbury, MD, USA; University of Missouri Health Care, Columbia, MO, USA; Great Plains Health, North Platte, NE, USA; Richmond University Medical Center, Staten Island, NY, USA; Brody School of Medicine at East Carolina University, Greenville, NC, USA; University of Toledo Medical Center, Toledo, OH, USA; Memorial Hermann Hospital Affiliated with the University of Texas Health Science Center at Houston, McGovern Medical School, Houston, TX, USA; and University of Utah Health, Salt Lake City, UT, USA.

## **Key Eligibility Criteria**

### *Inclusion Criteria*

1. Patients aged ≥18 years
2. Patients (or legally authorized decision maker) must provide informed consent form
3. Test positive for SARS-CoV-2 virus by PCR (positive confirmation prior to start of sargramostim)
4. Admitted to hospital
5. Presence of acute hypoxemic respiratory failure defined as (either or both)
   1. Saturation below 93% on ≥2 L/min oxygen supplementation
   2. PaO2/FiO2 below 350

### *Exclusion Criteria*

1. Patients requiring invasive (mechanical ventilation) or non-invasive (CPAP, BiPAP for hypoxemia) ventilation or ECMO. (Note: oxygen supplementation using high flow oxygen systems or low flow oxygen systems would not exclude patients from this study)
2. Intractable metabolic acidosis
3. Cardiogenic pulmonary edema
4. Hypotension requiring use of vasopressors
5. Hyperferritinemia (serum ferritin ≥2,000 mcg/L)
6. White blood cell count >50,000/mm3
7. Participation in another interventional clinical trial for COVID-19 therapy
8. Highly immunosuppressive therapy or anti-cancer combination chemotherapy within 24 hours prior to first dose of sargramostim
9. Known or suspected intolerance or hypersensitivity to sargramostim, or any component of the product
10. Previous experience of severe and unexplained side effects during aerosol delivery of any kind of medical product
11. Presence of any preexisting illness that, in the opinion of the Investigator, would place the patient at an unreasonably increased risk through participation in this study.
12. Pregnant or breastfeeding females

## **Procedures**

Assessments and examinations were performed according to institutional standard clinical practice for managing COVID-19 patients and included physical examinations, vital sign measurement, oxygen saturation measurements, electrocardiogram, clinical laboratory tests, chest x-ray, and computed tomography. Trial-specific assessments included measurement of serum ferritin, d-dimer, and c-reactive protein, arterial blood gases (ABG), and documentation of supplemental oxygen, ventilatory support, and adverse events. Oxygenation parameters were measured by pulse oximetry and ABG, and were corrected for altitude, where necessary.

## **Outcomes**

### *P(A-a)O_2_*

P(A-a)O_2_ measures the difference between the oxygen concentration in the alveoli and arterial system and can help determine the underlying cause for hypoxemia. P(A-a)O_2_ is derived from the arterial oxygen and carbon dioxide pressures and is used to detect ventilation/perfusion abnormalities. The P(A-a)O_2_ is computed as the difference between the alveolar oxygen partial pressure (PAO_2_; calculated as the fraction of oxygen inspired (FiO2) x (atmospheric pressure - water pressure) – partial pressure of carbon dioxide (PaCO_2_)/0.8) and the arterial oxygen pressure (PaO_2_).

### *Ordinal Scale*

The ordinal score recognizes multiple clinically meaningful dispositions between disease cure and death. Easily observed parameters, including hospitalization, activities, and oxygen / ventilatory support enable simple assessment. The score is non-binary and can be measured daily or at specific timepoints, providing potential ability to differentiate disease activity in the 2 treatment groups. Ordinal scores based on corresponding clinical status are detailed below.

**Ordinal Scale**

| **Measure** | **Ordinal Score** |
| --- | --- |
| Death | 8 |
| Hospitalized, mechanical ventilation + additional organ support (i.e., requiring ECMO, pressors, renal replacement therapy) | 7 |
| Hospitalized, requiring intubation and mechanical ventilation | 6 |
| Hospitalized, requiring high-flow oxygen therapy, non-invasive mechanical ventilation, or both | 5 |
| Hospitalized, requiring supplemental oxygen by mask or nasal prongs | 4 |
| Hospitalized, not requiting supplemental oxygen | 3 |
| Not hospitalized, limitation of activities | 2 |
| Not hospitalized, no limitation of activities | 1 |
| Not hospitalized, no clinical or virological evidence of infection | 0 |

### *Additional Planned Outcomes Not Reported*

Additional planned outcome measures that were analyzed but not reported here due to limited data collection include secondary outcome measures of time to improvement in oxygenation, rate of nosocomial infections and time to normalization of WBC and lymphocytes; and exploratory outcome measures of NEWS-2, SOFA and ROX index scores, progression to ARDS and changes on chest X-ray/CT.

## **Statistical Analysis**

The analytic study period began at the date/time of first dose for patients who received sargramostim. For patients who received standard of care (SOC) alone, randomization date/time was used as the start of the analytic period. Time to event endpoints were defined as start date/time (as defined above) to end date/time. Censoring dates were the last date/time the patient was documented to be event free. Depending on the sargramostim first dose timing, the last planned dose could be on the afternoon/evening of day 5 or the morning/afternoon of day 6. Therefore, the up-to-day 6 evaluation for either treatment arm could include assessments performed on day 7. For the sargramostim arm, an ABG assessment taken within one day of the last sargramostim dose was used. For SOC arm, an ABG assessment taken within 1 day of the treatment completion/discontinuation/Day 6 visit date was used. If there were no assessments in that timeframe, last post-baseline observation prior to treatment completion/discontinuation/Day 6 visit date was used.

The percent change in endpoints, such as the P(A-a)O_2_, were calculated for each patient as the change from baseline divided by the baseline value. Then, summary statistics were generated.

# **Supplementary Results**

## **Duration of Supplemental Oxygen**

No differences were observed between arms in duration of supplemental oxygen. Mean (SD) duration of any oxygen support was 37.9 (35.6) days in the sargramostim arm compared to 32.8 (34.3) days in the SOC arm (*P* = 0.44). Median [IQR] duration of any oxygen support was 26.0 [6.0, 77.0] days on the sargramostim arm and 18.0 [6.0, 52.0] days on the SOC arm.

## **Hematologic Data**

Hematology assessments were obtained according to the institutional SOC. Different institutions decided whether to conduct hematology laboratory assessments based on resources and clinical relevance of the test.

Most hematology parameter abnormalities were grade 1 to grade 2 in severity. There were no excess incidence of grade 3 or grade 4 severity observed post-baseline. Change from baseline neutrophil/lymphocyte ratio data were insufficient for analysis or interpretation. No clinically meaningful differences were noted between study arms.

## **Table S1: Availability of P(A-a)O_2_ Assessments by Study Day**

| **Parameter** | **Sargramostim arm**  **(n = 78)** | **SOC** **arm**  **(n = 44)** | **Total**  **(N = 122)** |
| --- | --- | --- | --- |
| Patients with Baseline P(A-a)O_2_ measurements, *n* (%) | 71 (89.7) | 41 (93.2) | 112 (91.8) |
| **Patients Included in Primary Endpoint Analysis (had Baseline and Post-Baseline P(A-a)O_2_ measurements), *n* (%)** | **63 (80.8)** | **33 (75.0)** | **96 (78.7)** |
| Post-Baseline Assessed on Day 1, n | 1 | 0 | 1 |
| Post-Baseline Assessed on Day 2, n | 1 | 1 | 2 |
| Post-Baseline Assessed on Day 3, n | 4 | 1 | 5 |
| Post-Baseline Assessed on Day 4, n | 4 | 2 | 6 |
| Post-Baseline Assessed on Day 5, n | 13 | 5 | 18 |
| Post-Baseline Assessed on Day 6, n | 37 | 20 | 57 |
| Post-Baseline Assessed on Day 7, n | 3 | 4 | 7 |
| **Patients Not Included in Primary Endpoint Analysis, *n* (%)** | **15 (19.2)** | **11 (25.0)** | **26 (21.3)** |

*Definition of abbreviations*: P(A-a)O_2_ *=* alveolar-arterial oxygen gradient; SOC *=* standard of care.

## **Table S2: Safety Parameters for Markers of Inflammation**

| **Parameter** | **Sargramostim arm**  **(n = 78)** | **SOC arm**  **(n = 44)** |
| --- | --- | --- |
| Ferritin, mcg/L |  |  |
| Baseline, *n* | 77 | 44 |
| Mean (SD) | 609.0 (448.5) | 700.9 (584.4) |
| Median [IQR] | 544.0 [287.6, 828.4] | 522.9 [229.0, 921.1] |
| Day 6 change from baseline, *n* | 48 | 29 |
| Mean (SD) | -43.2 (492.5) | -15.5 (401.2) |
| Median [IQR] | -77.9 [-166.5, 24.0] | -61.0 [-162.0, 78.0] |
| D-dimer, nmol/L |  |  |
| Baseline, *n* | 68 | 43 |
| Mean (SD) | 7.1 (14.4) | 4.1 (5.3) |
| Median [IQR] | 2.2 [1.1, 5.2] | 1.6 [1.1, 4.8] |
| Day 6 change from baseline, *n* | 39 | 28 |
| Mean (SD) | -0.1 (9.0) | 0.5 (5.5) |
| Median [IQR] | -0.1 [-2.5, 0.7] | -0.6 [-2.7, 0.6] |
| C-reactive protein, mg/L |  |  |
| Baseline, *n* | 68 | 41 |
| Mean (SD) | 83.5 (60.6) | 100.0 (83.1) |
| Median [IQR] | 64.1 [42.7, 109.6] | 71.1 [44.5, 129.5] |
| Day 6 change from baseline, *n* | 42 | 24 |
| Mean (SD) | -43.5 (71.6) | -50.6 (109.4) |
| Median [IQR] | -32.5 [-72.4, -1.5] | -27.7 [-100.1, -4.2] |

*Definition of abbreviations*: IQR = interquartile range; SOC *=* standard of care.

Patients evaluated at both baseline and post-baseline timepoint are included in the table for change from baseline. Number of patients evaluated are noted for each outcome.

## **Figure S1: Percent Change in P(A-a)O_2_ from Baseline (Y-axis) to Up-to-day 6 Evaluation for Each Patient (X-axis)**

**
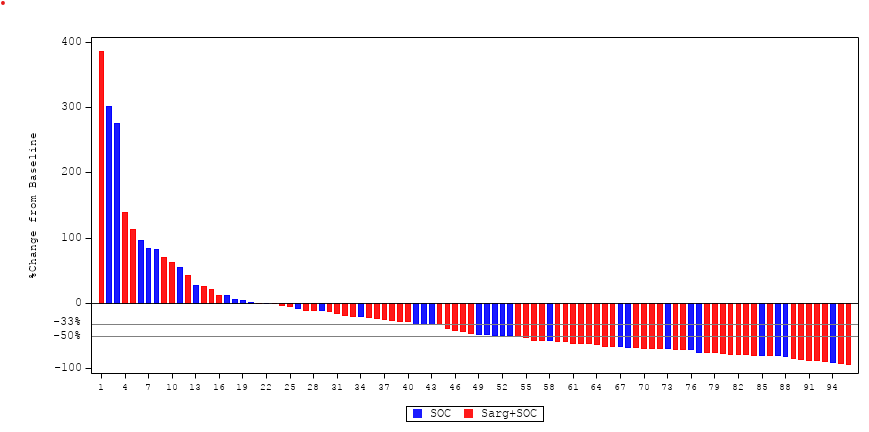
**

*Definition of abbreviations*: P(A-a)O_2_ *=* alveolar-arterial oxygen gradient; Sarg *=* sargramostim; SOC *=* standard of care.
