# Supplementary material for: Inhaled Sargramostim (Recombinant Human Granulocyte-Macrophage Colony-Stimulating Factor) for COVID-19-Associated Acute Hypoxemia: Results of the Phase 2, Randomized, Open-Label Trial (iLeukPulm)
Source: Mil Med. 2022 Dec 2;188(7-8):e2629–38. doi: 10.1093/milmed/usac362 (PMC10363010; doi:10.1093/milmed/usac362)
Supplement: usac362_Supp [file usac362_supp.zip › iLeukPulm_MilMed_GraphicalAbstract_20220728.pptx]

## Slide 1
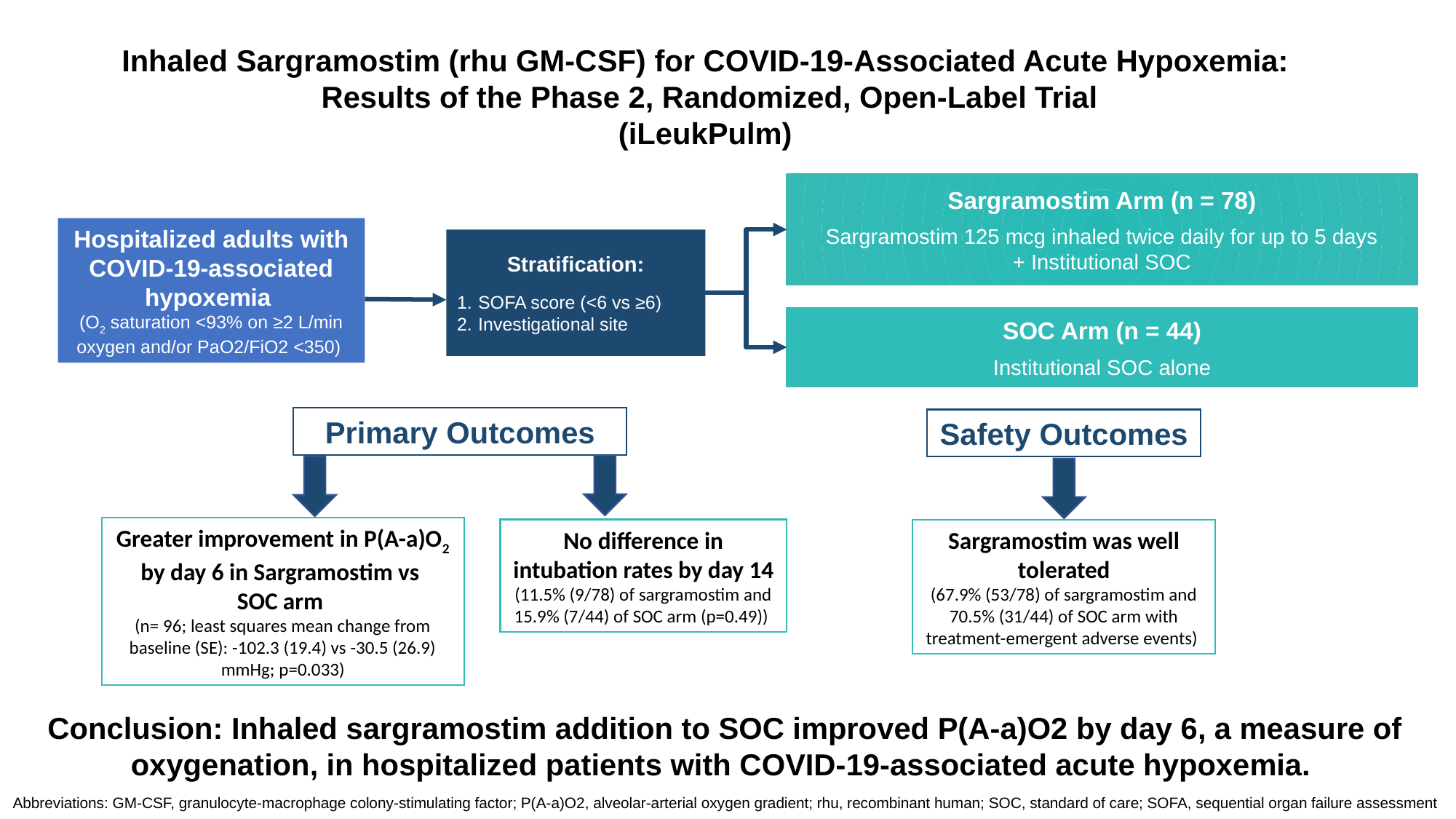

Inhaled Sargramostim (rhu GM-CSF) for COVID-19-Associated Acute Hypoxemia:
 Results of the Phase 2, Randomized, Open-Label Trial
(iLeukPulm)
Sargramostim Arm (n = 78)
Sargramostim 125 mcg inhaled twice daily for up to 5 days
+ Institutional SOC
Stratification:
SOFA score (<6 vs ≥6)
Investigational site
SOC Arm (n = 44)
Institutional SOC alone
Hospitalized adults with COVID-19-associated hypoxemia
(O2 saturation <93% on ≥2 L/min oxygen and/or PaO2/FiO2 <350)
Primary Outcomes
Safety Outcomes
Greater improvement in P(A-a)O2 by day 6 in Sargramostim vs
SOC arm
(n= 96; least squares mean change from baseline (SE): -102.3 (19.4) vs -30.5 (26.9) mmHg; p=0.033)
No difference in intubation rates by day 14
(11.5% (9/78) of sargramostim and 15.9% (7/44) of SOC arm (p=0.49))
Sargramostim was well tolerated
(67.9% (53/78) of sargramostim and 70.5% (31/44) of SOC arm with treatment-emergent adverse events)
Conclusion: Inhaled sargramostim addition to SOC improved P(A-a)O2 by day 6, a measure of oxygenation, in hospitalized patients with COVID-19-associated acute hypoxemia.
Abbreviations: GM-CSF, granulocyte-macrophage colony-stimulating factor; P(A-a)O2, alveolar-arterial oxygen gradient; rhu, recombinant human; SOC, standard of care; SOFA, sequential organ failure assessment
